# Supplementary figures and images for: Effects of different training modalities on phosphate homeostasis and local vitamin D metabolism in rat bone
Source: PeerJ. 2019 Jan 24;7:e6184. doi: 10.7717/peerj.6184 (PMC6348094; doi:10.7717/peerj.6184)

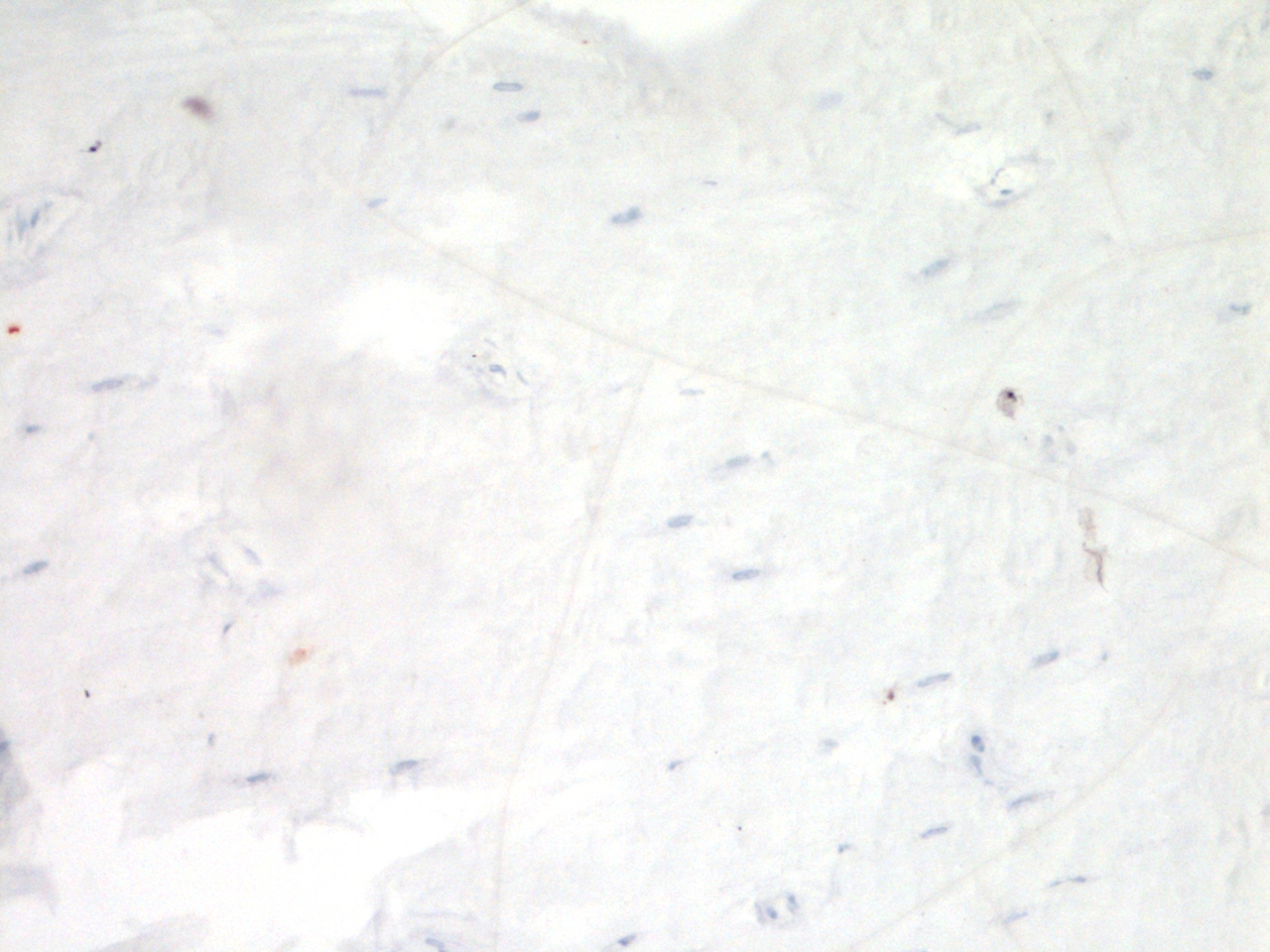

Supplement: Supplemental Information 2 [file peerj-07-6184-s002.jpg]

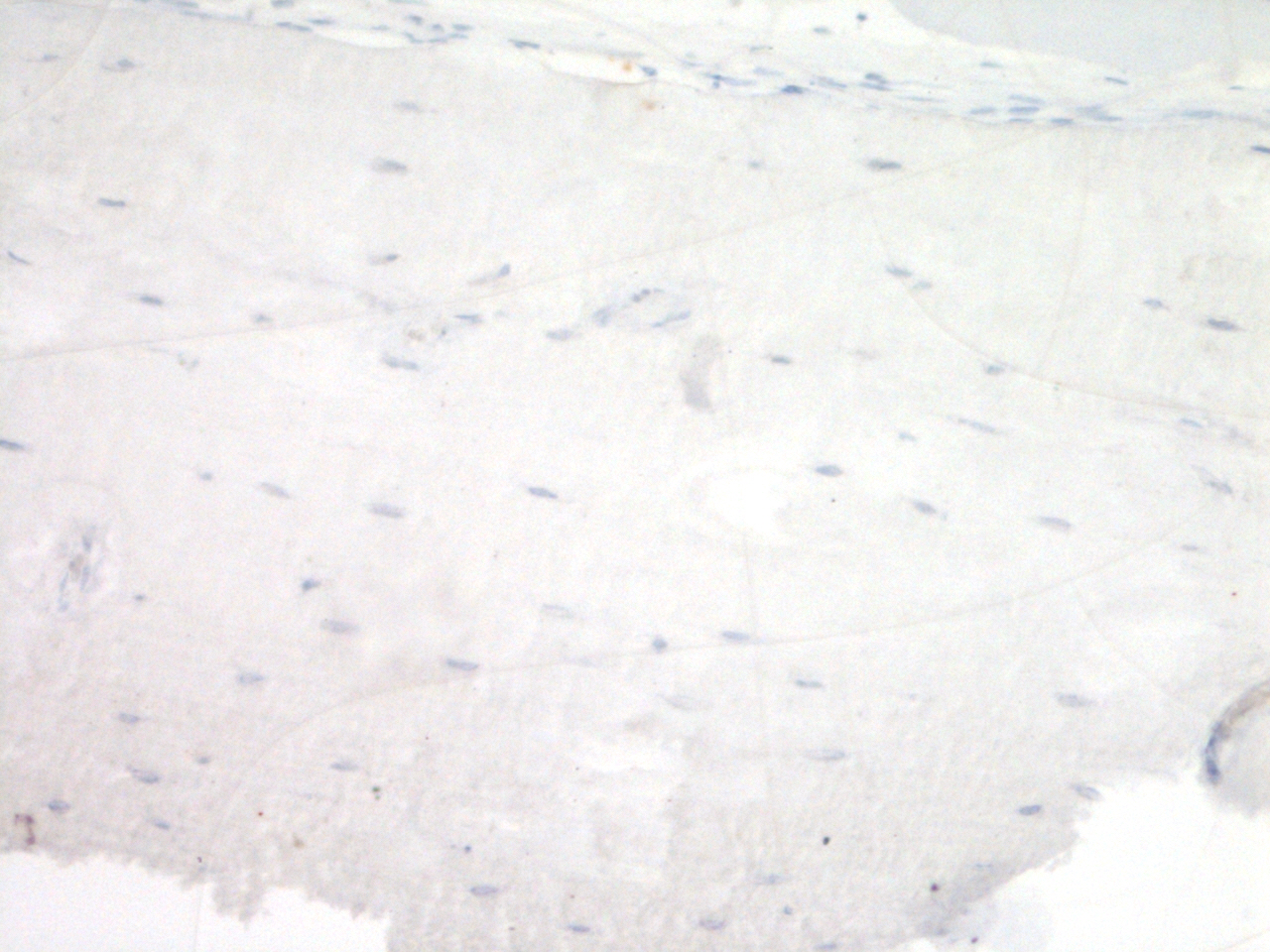

Supplement: Supplemental Information 3 [file peerj-07-6184-s003.jpg]
